# Long-Term Mild, rather than Intense, Exercise Enhances Adult Hippocampal Neurogenesis and Greatly Changes the Transcriptomic Profile of the Hippocampus

**DOI:** 10.1371/journal.pone.0128720

**Published:** 2015-06-10

**Authors:** Koshiro Inoue, Masahiro Okamoto, Junko Shibato, Min Chul Lee, Takashi Matsui, Randeep Rakwal, Hideaki Soya

**Affiliations:** 1 Laboratory of Exercise Biochemistry & Neuroendocrinology, Faculty of Health and Sport Sciences, University of Tsukuba, Tsukuba, Ibaraki, 305–8574, Japan; 2 School of Rehabilitation Science, Health Sciences University of Hokkaido, Kanazawa, Ishikari-Tobetsu, Hokkaido, 061–0293, Japan; 3 Department of Anatomy, Showa University School of Medicine, Shinagawa, Hatanodai, Tokyo, 142–8555, Japan; 4 Japan Society for the Promotion of Science, Tokyo, Japan; 5 Organization for Educational Initiatives, University of Tsukuba, Tsukuba, 305–8577, Ibaraki, Japan; University of Amsterdam, NETHERLANDS

## Abstract

Our six-week treadmill running training (forced exercise) model has revealed that mild exercise (ME) with an intensity below the lactate threshold (LT) is sufficient to enhance spatial memory, while intense exercise (IE) above the LT negates such benefits. To help understand the unrevealed neuronal and signaling/molecular mechanisms of the intensity-dependent cognitive change, in this rat model, we here investigated plasma corticosterone concentration as a marker of stress, adult hippocampal neurogenesis (AHN) as a potential contributor to this ME-induced spatial memory, and comprehensively delineated the hippocampal transcriptomic profile using a whole-genome DNA microarray analysis approach through comparison with IE. Results showed that only IE had the higher corticosterone concentration than control, and that the less intense exercise (ME) is better suited to improve AHN, especially in regards to the survival and maturation of newborn neurons. DNA microarray analysis using a 4 × 44 K Agilent chip revealed that ME regulated more genes than did IE (ME: 604 genes, IE: 415 genes), and only 41 genes were modified with both exercise intensities. The identified molecular components did not comprise well-known factors related to exercise-induced AHN, such as brain-derived neurotrophic factor. Rather, network analysis of the data using Ingenuity Pathway Analysis algorithms revealed that the ME-influenced genes were principally related to lipid metabolism, protein synthesis and inflammatory response, which are recognized as associated with AHN. In contrast, IE-influenced genes linked to excessive inflammatory immune response, which is a negative regulator of hippocampal neuroadaptation, were identified. Collectively, these results in a treadmill running model demonstrate that long-term ME, but not of IE, with minimizing running stress, has beneficial effects on increasing AHN, and provides an ME-specific gene inventory containing some potential regulators of this positive regulation. This evidence might serve in further elucidating the mechanism behind ME-induced cognitive gain.

## Introduction

Although dependent upon intensity based on lactate threshold (LT; approximately 50 to 60% of maximal oxygen consumption in humans), exercise has a positive effect on the function of the hippocampus, a brain region that plays an important role in the formation of memory [1–5]. The LT is known as a reference point where the steady state of blood lactate accumulation breaks down during incremental exercise. At intensities above the LT, exercise causes a stress response characterized by a slight rise in the levels of adrenocorticotropic hormone and corticosterone (CORT) in the blood and by activation of hypothalamic loci, known as a center of homeostatic regulation including the stress response [6–8]. Therefore, based on the LT, exercise is separated into mild exercise (ME; <LT), which can be described as a stress-free exercise condition in our study, or intense exercise (IE; >LT), which is accompanied by exercise-derived stress [9]. ME yields greater benefits for hippocampal function than does IE; our recent study showed that rats that underwent six weeks of mild (ME), but not intense (IE), treadmill running training had increased spatial memory in a Morris water maze (MWM) compared to sedentary controls (CONT) [5]. However, the regulatory mechanism remains unclear.

Exercise increases adult hippocampal neurogenesis (AHN) [1,10–12], that is a continuous production of new neurons in the hippocampal dentate gyrus (DG) [13–15], and furthermore, exercise-enhanced AHN is considered an important cellular substrate for the development of hippocampal function [1,4,10]. The origin of AHN is adult neural stem cells, which can grow into functionally matured neurons through a development process that is divided into three main stages (cell proliferation, differentiation and functional maturity) over four to six weeks [13–15]. The newly generated mature neurons are considered to be a contributor in memory formation because of having a functional synaptic connection with the existing neuronal circuit and the unique morphological features of dendritic spine for enhancing synaptic potentiation [16–19]. The evidences showing that the neurons are preferentially activated during hippocampal-dependent behavioral task [13–15,20] also support their important contribution to memory processing. Most importantly, a study by Clark *et al*. (2008) found that the suppression of exercise-induced AHN by irradiation eliminates the enhanced hippocampal function, especially the increased spatial memory, as revealed by the MWM task [21]. Though the role of AHN in hippocampal function is a debatable issue [13–15,22], these findings imply that the exercise-intensity-dependent effect on spatial memory may be related to the difference of AHN, especially of the number of mature neurons, in each exercise condition.

In our laboratory, we have investigated exercise-intensity-dependent changes in AHN for a two-week treadmill running training model, and confirmed that ME increases AHN, while these effects disappear with IE [23]. Althought the results demonstrate that ME is more beneficial for increasing AHN than is IE, this cannot be easily translated to a six-week exercise model since animals subjected to long-term intense exercise sometimes exhibit a stress adaptation [24] and resistance to environmental stresses [25]. If IE causes the stress adaptation within the six weeks of the training period, it might lead to increased AHN with no change in the plasma levels of stress-related hormones, such as CORT, a negative regulator of AHN [26]. Furthermore, a longer period of exercise training seems to be required to increase the mature neuron associated to memory formation. In fact, previous studies examining the effects of exercise in AHN have shown that long-term (≧ 4 weeks) exercise increases the number of cells that differentiate into neurons, enhancing synaptic plasticity in the DG [11,12], and increasing hippocampal function in parallel [1]. Other studies have interestingly reported that the new neurons generated in response to exercise are integrated into functional hippocampal circuitry that could respond to exercise as well as spatial learning and memory at five to seven weeks [27,28]. These finding suggest that at least over four weeks of exercise training and the exercise-induced production of new neurons play an important role in enhancing spatial memory, and require the investigation of AHN in our six-week exercise model [5]. Considering that six weeks of IE increased plasma CORT levels compared to the sedentary control [5], the six weeks of ME, but not of IE, may increase the AHN, especially the number of mature neurons that contribute to the improvement of the spatial memory.

In addition, numerous factors are assumed to be potential regulators of ME-induced increases in AHN. For example, our past study revealed that ME-induced AHN is promoted through androgenic mediation [23]. In contrast, other researchers have shown that brain-derived neurotrophic factor (BDNF), insulin growth factor (IGF1) and vascular endothelial growth factor (VEGF), and their respective signaling pathways seem to be of special importance in regulating exercise-induced AHN. BDNF up-regulation following exercise is associated with a robust activation of survival pathways to enhance AHN [4,29,30]. Circulatory IGF1 or VEGF have also been reported as essential factors in exercise-induced AHN [31–33]. Other factors, such as progranulin [34], steroids (glucocorticoid [35]), morphogens (Wnt [36]) and neurotransmitters (glutamate [37], neuropeptide Y [38] and beta-endorphin [39]), have also been assumed to be involved in these adaptations. Collectively, this evidence suggests that exercise promotes AHN through a complex regulatory mechanism, and numerous factors, including androgen, are potentially involved in ME-induced AHN.

To achieve a deeper insight into the complex molecular mechanism, a comprehensive omics approach, such as the genome-wide transcriptome profiling, would be indispensable. DNA microarray technology is a high-throughput, powerful approach to the rapid screening and quantification of differences in gene expression between large groups [40]. Its technical features help us to systematically realize the complex process of exercise-induced hippocampal gene alteration. Previous studies have widely used this technology to delineate the spectrum of exercise-inducible gene expression, and have indicated that exercise, especially voluntary exercise, initiates the expression of genes related to synaptic and protein trafficking, the glutamatergic system, neural structure, neurotrophins and neuropeptides [41–44]. While these include genes unrelated to AHN, data interpretation focused on AHN-related genes, such as Burger’s study [45], provides insight into the hierarchical importance of genes and signaling pathways associated with exercise-induced AHN.

Hence, the aim of this paper is to explore the mechanisms behind why ME is more suitable than IE for enhancing spatial memory in six-week of treadmill running training model. To do so, we investigated the exercise intensity-dependent change in the AHN and in the hippocampal transcriptome genome-wide using the previous model. We first checked the plasma CORT levels, and tested the hypothesis that ME, but not IE, could enhance AHN, especially the number of new mature neurons, compared with a sedentary control group. Finally, the ME- and IE-related gene alterations potentially linked with AHN was delineated using a rat whole genome 4 × 44 K Agilent DNA microarray chip.

## Materials and Methods

### Animals and ethics statement

Eleven-week-old male Wistar rats (220–270 g; Japan SLC) were housed in polycarbonate steel cages with a 12-h light/dark schedule (lights on at 7:00 a.m.) and given *ad libitum* access to food and water. A total of 44 rats were used in this study to assess the influence of exercise intensity on AHN (n = 22) and hippocampal transcriptome by DNA microarray (n = 22). All animal care and experimental procedures were performed in accordance with protocols approved by the University of Tsukuba Animal Experiment Committee, based on the National Institute of Health (NIH) Guidelines for the Care and Use of Laboratory Animals (NIH publication, revised 1996). Every effort was made to minimize animal suffering and to reduce the number of animals used in this study. To adapt to the rearing environment and the experimenter, and remove any unintended stress effects, animals underwent a week of preliminary rearing to ambient conditions with gentle handling in group housing (2–3 rats/cage).

### Exercise training procedure

The apparatus and training protocol were similar to those previously described [5]. Briefly, after preliminary rearing, the animals were randomly divided into three groups, which were based on LT for treadmill running [6,7]: sedentary control (CONT, at rest on the treadmill), mild exercise (ME, below LT, 15 m/min) and intense exercise (IE, above LT, 40 m/min) with no incline. Exercise groups were habituated to treadmill (KN-73; Natsume) running for one (ME) or four (IE) weeks by using slight electric shocks. Within their respective habituation periods, rats were able to run at the appropriate speeds even when the electric grid was turned off. It is to be noted that the ME rats were assigned the exercise training without electrical stimulation excluding the one-week of running habituation, as shown in our previous study [8]. Certain IE rats, on the other hand, sometimes refused to run at the set treadmill speed after the running habituation period, and if so, further foot shocks were sparingly given to them to motivate their running. To minimize any stress derived from the shocks that might have hindered any of the beneficial physiologic effects induced by exercise; any given electric shock was limited to < 1 sec duration when rats did not restart the running with a gentle tail touch. It is to be noted that any of those shocks were limited to within the adaptable range [46,47], and the acceptable range [48,49] of the amount of electrical shocks to avoid any further non-specific stress. Among the 16 IE rats, four rats that refused to run were finally excluded, as shown previously [50]. AHN-group rats were injected intraperitoneally with 5-bromo-2'-deoxyuridine (BrdU; Nacalai Tesque) (100 mg/kg BW) once a day for three consecutive days at the onset of running habituation. All rats trained for 6 weeks in total including the habituation period. Animals ran for 60 min/day (except for the first day, on which they ran for 30 min), five times/week between 19:00 and 22:00 (during the dark phase). The daily running time included a warm-up phase to prepare rats for running at the set speed. The protocol was exactly as described in a previous study [5], and is delineated in S1 Table. Physical condition and body weight were monitored until the end of the training period. If rats exhibited poor health or did not vigorously run at their determined speed, they were excluded from the study. Two days after the last training session, rats were advanced to the next step of the experiment.

### Tissue preparation

In the AHN group, rats were anaesthetized with a lethal volume of sodium pentobarbital, and transcardially perfused with 0.9% saline followed by 4% paraformaldehyde (PFA) in 0.1 M phosphate buffer. Brains were removed, fixed overnight in PFA and then transferred to 20% sucrose at 4°C. Coronal sections of 50 μm were serially cut through the entire rostral caudal extent of dentate gyrus (DG) (-1.5 to -6.5 mm from bregma) using a cryostat (HM505E; MICROM), and stored at -30°C until they were processed for antibody reaction.

In the microarray group, immediately after decapitation, trunk blood was quickly sampled to measure physiological stress levels based on plasma CORT, and the whole brain of each animal was rapidly removed. The hippocampus was separated from the brain on ice according to the method by Hori *et al*. [51], and immediately flash-frozen in liquid nitrogen. The procedures were performed in the morning (08:00–11:00). The deep-frozen hippocampus was then transferred to a pre-chilled (in liquid nitrogen) mortar and pestle, and ground to a very fine powder with liquid nitrogen. The trunk blood and the powdered samples were stored in aliquots at -80°C until further analysis.

### Plasma corticosterone measurement

Plasma CORT levels were measured with radioimmunoassay as described previously [52,53]. For competitive binding, we utilized [3H]-CORT (Tokyo Chemical Industry) and anti-corticosterone antibody (Cosmo Bio). After 16 hours, anti-CORT bound CORT was separated from unbound CORT by adding 500 μl of Dextran-coated charcoal. The samples were centrifuged at 4°C (3,000 rpm, 10 min) and immediately transferred to scintillation vials filled with 4 ml of a liquid scintillation cocktail (Clear-sol II; Nacalai Tesque). The vials were briefly shaken followed by a count of the CORT concentration with a LS6000 Beckman scintillation counter (Beckman Coulter). The detection limit of the CORT assay was 0.2 ng/dl. The inter-assay coefficients in the variation of CORT were 12%. Final plasma CORT concentration was determined by the average of its duplicate concentrations.

### Immunohistochemical and stereological analysis of AHN

Immunohistochemical staining and stereological counting of the antibody-labeled cells was carried out as described previously [23]. Briefly, the target cells were stained with appropriate antibodies on every 10^th^ unilateral section (10–11 sections per rat), and the immunolabeled cells in the subgranular zone (SGZ) and the granule cell layer (GCL) of the DG were counted [33,54]. Based on this number, the total number of labelled cells was calculated by multiplication of the mean cell density in all sections by the total DG volume. The total DG volume was estimated by multiplying surface area of DG (SGZ and GCL) by thickness of the section (50 μm) following the Cavalieri method [55]. The surface area was made visible with Nissl staining, and quantified using the image analysis software ImageJ ver. 1.44 (NIH, http://rsb.info.nih.gov/ij). For immunohistochemical staining, a 1-in-10 series of sections was randomly selected, and preincubated in 0.1 M phosphate buffer containing 1% Triton X-100 (PBT). The free-floating slices were incubated in 2 N HCl at 37°C for 30 min to denature the DNA, and then soaked with 2% (vol/vol) normal donkey serum in PBT (2% NDS-PBT) for 30 min to block nonspecific binding of antibodies. The slices were then well rinsed with PBT, and incubated for 2 d at 4°C with the primary antibodies diluted with 2% NDS-PBT containing 1% BSA. Rat anti-BrdU (AbD Serotec; 1: 500), a newly born cell marker, and mouse anti-NeuN (Chemicon; 1:500), a mature neuron marker, were used as the primary antibodies. After washing the primary antibody with PBT, the slices were incubated to visualize the antigens for 24 h at 4°C with secondary antibodies as follows: Cy3-conjugated donkey anti-rat IgG (Jackson ImmunoResearch Laboratories; 1: 500), and AMCA or Alexa Fluor 488 conjugated donkey anti-mouse IgG (Jackson ImmunoResearch Laboratories; 1: 250 or Life Technologies; 1: 500). Another series of sections were rinsed in PBT, and then soaked in PBT containing 0.3% H_2_O_2_ for 30 min to quench endogenous peroxidase activity. The sections were well washed with PBT, and incubated with blocking solution containing 2% (vol/vol) normal goat serum in PBT (2% NGS-PBT) for 60 min. The sections were then incubated in rabbit anti-Ki67 primary antibody (Abcam; 1:1000), a marker of cells undergoing cell cycle activation, diluted with 2% NGS-PBT for 24 h at 4°C. After incubation, the antigens were visualized using a modified avidin-biotin-peroxidase complex (ABC) method, as described previously [8]. With the ABC method, the sections incubated in biotinylated rabbit IgG (1:200; Vectastain Elite ABC Kit, Vector Laboratories) diluted with 2% NGS-PBT for 2 h. The sections were then well rinsed in PBT, and soaked with ABC solution (1:50; Vectastain Elite ABC Kit) for 90 min following manufacturer’s instructions. After rinsing with PBT and 0.1 M acetate buffer, the antigens were rendered visible using a solution of 0.025% 3,3-diaminobenzidine tetrahydro- chloride, 0.08% ammonium chloride, 0.4% glucose, and 0.03% glucose oxidase (10,000 IU) in PBT for 5–10 min at room temperature. Finally, the sections were stained using Nissl staining to identify the DG area. For Nissl staining, the series of brain slices were mounted on gelatin-coated slides and air-dried. The dried sections were stained with toluidine blue, dehydrated in a graded ethanol series (60–100%), delipidated in xylene, and coverslipped with Mount-Quick (Daido Sangyo). The cellular populations of proliferating cells (DAB-labeled Ki-67^+^ cells), surviving cells (BrdU^+^ cells), and newborn mature neurons (BrdU^+^/NeuN^+^ cells) throughout the DG were counted in unilateral sections using an optical microscope (DMRB; Leica).

### DNA microarray analysis

Total RNA was extracted from each powdered hippocampal sample (~50 mg) using the QIAGEN RNeasy Mini Kit (QIAGEN). To verify the quality of this RNA, the yield and purity were determined spectrophotometrically (NanoPhotomater; IMPLEN) and visually confirmed using formaldehyde gel electrophoresis. We only used samples with a ratio of spectrophotometric absorbance of 260 nm to 230 nm (A_260_/A_230_) or to 280 nm (A_260_/A_280_) above 1.8 (S2 Table). In each experimental group, an equal amount of RNA (1000 ng) from five randomly chosen rats was pooled and used for DNA microarray analysis. Total RNA (1000 ng) was labeled with either Cy3 or Cy5 dye using an Agilent Low RNA Input Fluorescent Linear Amplification Kit (Agilent Technologies). Fluorescently labeled targets of control as well as treated samples were hybridized to the same microarray slide with 60-mer probes (4 x 44K rat whole genome, G4131F, in total 41,090 gene probes, Agilent). A flip labeling (dye-swap or reverse labeling with Cy3 and Cy5 dyes) procedure followed to nullify the dye bias associated with unequal incorporation of the two Cy dyes into cDNA [51]. The use of a dye-swap approach provided a more stringent selection condition for changed gene expression profiling than a simple single- or two-color approach. Hybridization and wash processes were performed according to the manufacturer’s instructions, and hybridized microarrays were scanned using an Agilent microarray scanner G2565BA. For the detection of significantly differentially expressed genes between control and exercise samples each slide image was processed with Agilent Feature Extraction software (version 9.5.3.1). Briefly, (1) this program measured Cy3 and Cy5 signal intensities of whole probes; (2) dye-bias tends to be signal intensity dependent, and therefore the software selected probes using a set-by-rank consistency filter for dye normalization; (3) normalization was performed by LOWESS (locally weighted linear regression), which calculates the log ratio of dye-normalized Cy3 and Cy5 signals, as well as the final error of the log ratio; (4) the significance (P) value was based on the propagate error and universal error models; (5) the threshold of significance for differentially expressed genes was <0.01 (for the confidence that the feature was not differentially expressed); and (6) erroneous data generated owing to artifacts were eliminated before data analysis using the software. The outputs of microarray analysis used in this study are available under the series number GSE 45813 at the NCBI Gene Expression Omnibus (GEO) public functional genomics data repository (https://www.ncbi.nlm.nih.gov/geo/query/acc.cgi?acc=GSE45813).

### Confirmatory reverse transcriptase-polymerase chain reaction

To confirm the microarray data, reverse transcriptase polymerase chain reaction (RT-PCR), an accepted method for validating microarray results in the neuroscience field [56–59], was applied to randomly up- and down-regulated genes using 3`-UTR specific gene primers (S3 Table). Briefly, total RNA samples were first DNase-treated with RNase-free DNase (QIAGEN, Maryland). The reaction mixture contained 0.6 μl of the first-strand cDNA, 50 pmols of each primer set and 6.0 μl of the Emerald Amp PCR Master Mix (2X premix) (Takara Shuzo) in a total volume of 12 μl with sterile water supplied in the kit. Thermal-cycling (C1000 Thermal Cycler; Bio-Rad Laboratories) parameters were as follows: after an initial denaturation at 97°C for 5 min, samples were subjected to a cycling regime of 20 to 40 cycles at 95°C for 45 s, 55°C for 45 s, and 72°C for 1 min. At the end of the final cycle, an additional extension step was carried out for 10 min at 72°C. After completion of the PCR, the total reaction mixture was mixed, spun down, and loaded into wells of a 1.8% agarose gel (Nacalai Tesque). Electrophoresis was then performed for 22 min at 100 volts in 1X TAE buffer using a Mupid-ex electrophoresis system (ADVANCE). The gels were stained (8 μl of 10 mg/ml ethidium bromide in 200 ml 1X TAE buffer) for 7 min, and the stained bands were visualized using an UV-transilluminator (LAS-4000 mini; Fujifilm Life Science).

### Ingenuity Pathways Analysis

The functional and network analyses were generated through the use of IPA (Ingenuity Systems, www.ingenuity.com). The data set from the microarray, which was made up of the differentially expressed (≧/≦ 1.5/0.75-fold compared to CONT) genes, and the corresponding fold change values were uploaded as an Excel spreadsheet to the IPA tool. To create gene networks, the genes were overlaid onto a global molecular network developed from information contained in the Ingenuity Knowledge Base. The functional analysis identified the biological functions and/or diseases that were most significant to the data set (*p*<0.05) according to a right-tailed Fisher’s exact test.

### Statistical analysis

Data are expressed as mean ± SE. AHN data were compared using one-way ANOVA for comparisons between all groups using SPSS Statistical Package (SPSS). The Tukey method was applied for all other data as *post-hoc* analysis. Differences were considered statistically significant at *p*<0.05 by SPSS.

## Results

### Effect of exercise training on body weight and physiological stress level

**Fig 1:**
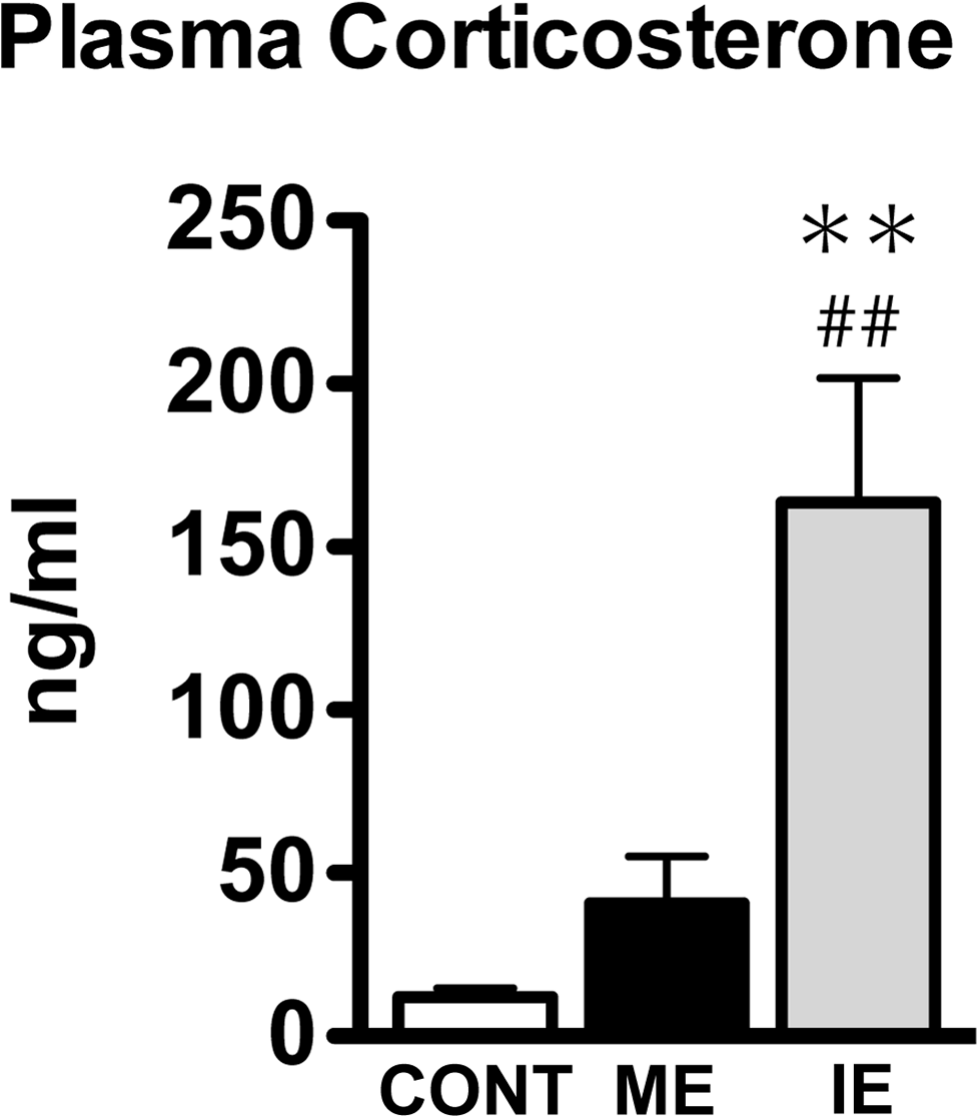
Exercise training effect on plasma corticosterone under different intensities of exercise. Effects of mild exercise (ME) or intense exercise (IE) on plasma corticosterone. Values are expressed as mean ± SE (n = 5). **p<0.01 compared to sedentary control (CONT) rats. ##*p*<0.01 compared to ME rats. Data were analyzed using a one-way ANOVA, followed by Tukey comparison *post-hoc* tests.

We first confirmed the body weight at the end of the training period for each group. ME (328.4 ± 4.45) and IE (315.8 ± 4.51) rats had significantly reduced body weights compared to CONT (348.8 ± 4.83; F (2,33) = 13.16, *p*<0.05), with no significant differences between the two exercise groups. Next, physiological stress levels were determined by examining changes in plasma CORT concentrations (ng/ml). The plasma CORT was significantly increased with IE (163.72 ± 38.03; F (2,12) = 11.72, *p*<0.05) compared to CONT (12.05 ± 2.65) and ME (40.45 ± 14.45), with no significant differences between ME and CONT (Fig 1).

### Adult hippocampal neurogenesis evaluation

**Fig 2:**
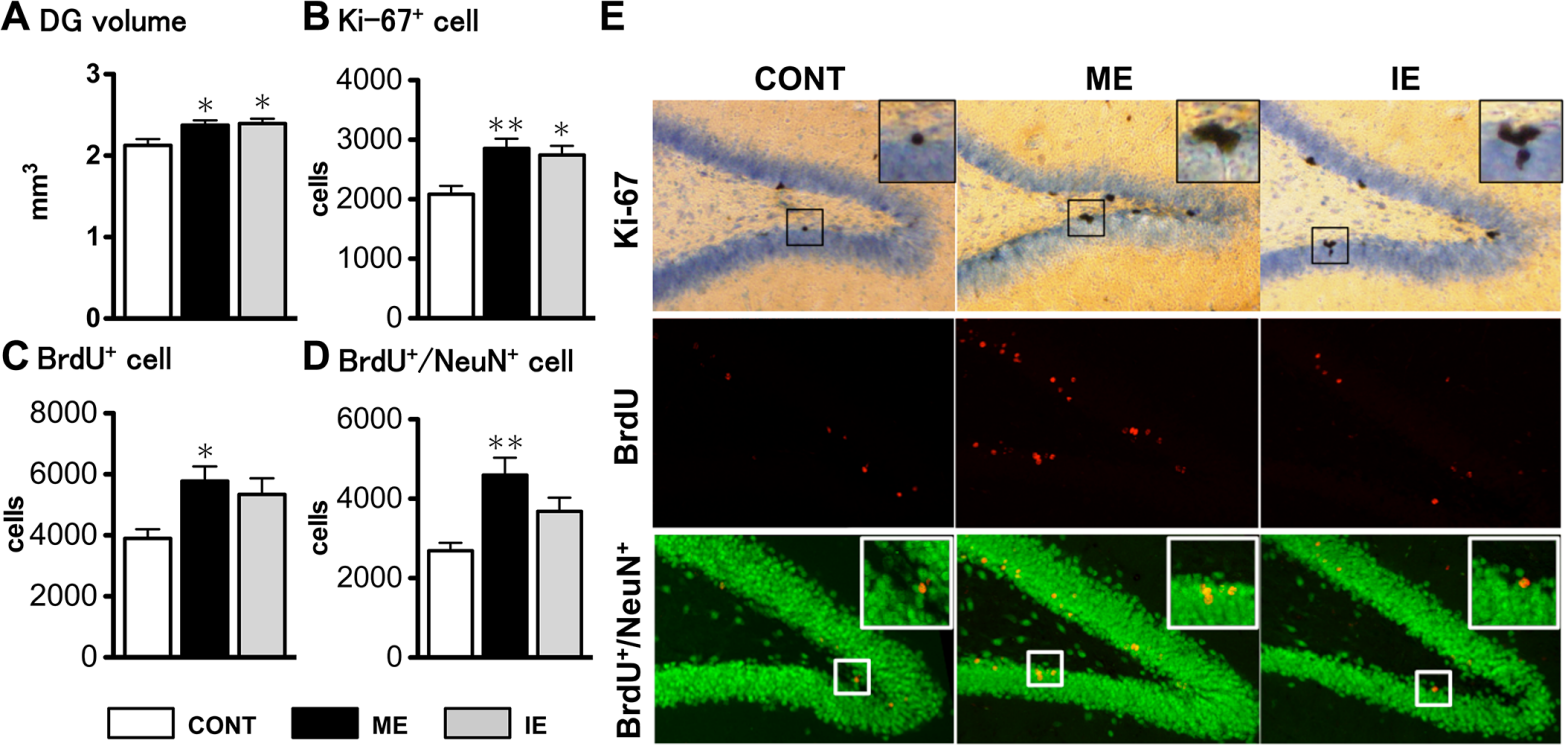
Hippocampal neuroanatomical changes induced by different intensities of exercise. Effects of mild exercise (ME) or intense exercise (IE) on hippocampal DG volume (A), the number of Ki-67^+^ cells (marker of neuronal progenitor proliferation) (B), BrdU positive cells (marker of cell survival) (C) and BrdU^+^/NeuN^+^ cells (marker of newly generate matured neurons) (D). Representative photograph of the dentate gyrus (DG) from each group single-stained for Ki-67 (brown), or double-stained for BrdU (red) and NeuN (green) (E). Values are expressed as mean ± SE (n = 7). * *p*<0.05; ** *p*<0.01 compared to sedentary control (CONT) rats. There is no significant difference between both exercise groups in all items. Data were analyzed using a one-way ANOVA, followed by Tukey comparison *post-hoc* tests.

A significant increase of DG volume was found in both exercise groups (ME: 2.37 ± 0.06; IE: 2.39 ± 0.06) compared with CONT (2.13 ± 0.08) regardless of intensity (F (2,18) = 4.96, *p*<0.05) (Fig 2A). Similarly, the number of Ki-67^+^ cells in the exercise groups (ME: 2852.7 ± 165.8; IE: 2742.4 ± 153.5; F (2,18) = 7.35, *p*<0.01) was significantly higher than that in the CONT group (2084.6 ± 139.1), with no significant difference between the two exercise intensities (Fig 2B). Conversely, exercise-intensity-dependent regulation was found in the total number of BrdU^+^ (F (2,18) = 4.82, *p*<0.05; Fig 2C) and BrdU^+^/NeuN^+^ (F (2,18) = 7.77, *p*<0.01; Fig 2D) cells. Further comparison with CONT (BrdU^+^: 3898.6 ± 300.9; BrdU^+^/NeuN^+^: 2691.4 ± 200.9) revealed that the cell numbers significantly increased in the ME group (BrdU^+^: 5775.1 ± 482.4; BrdU^+^/NeuN^+^: 4595.0 ± 438.3), but not in the IE group (BrdU^+^: 5343.9 ± 527.2; BrdU^+^/NeuN^+^: 3685.2 ± 342.6). The significant differences were not found between both exercise groups in the number of these cells (Fig 2C and 2D). For the BrdU^+^/NeuN^-^ cell numbers, there was no significant difference between any groups (CONT: 1207.2 ± 164.5; ME: 1180.0 ± 78.0; IE: 1675.1 ± 311.0; F (2,18) = 1.79, *p* = 0.20) (data not shown).

### Overview of the hippocampal transcriptome

**Fig 3:**
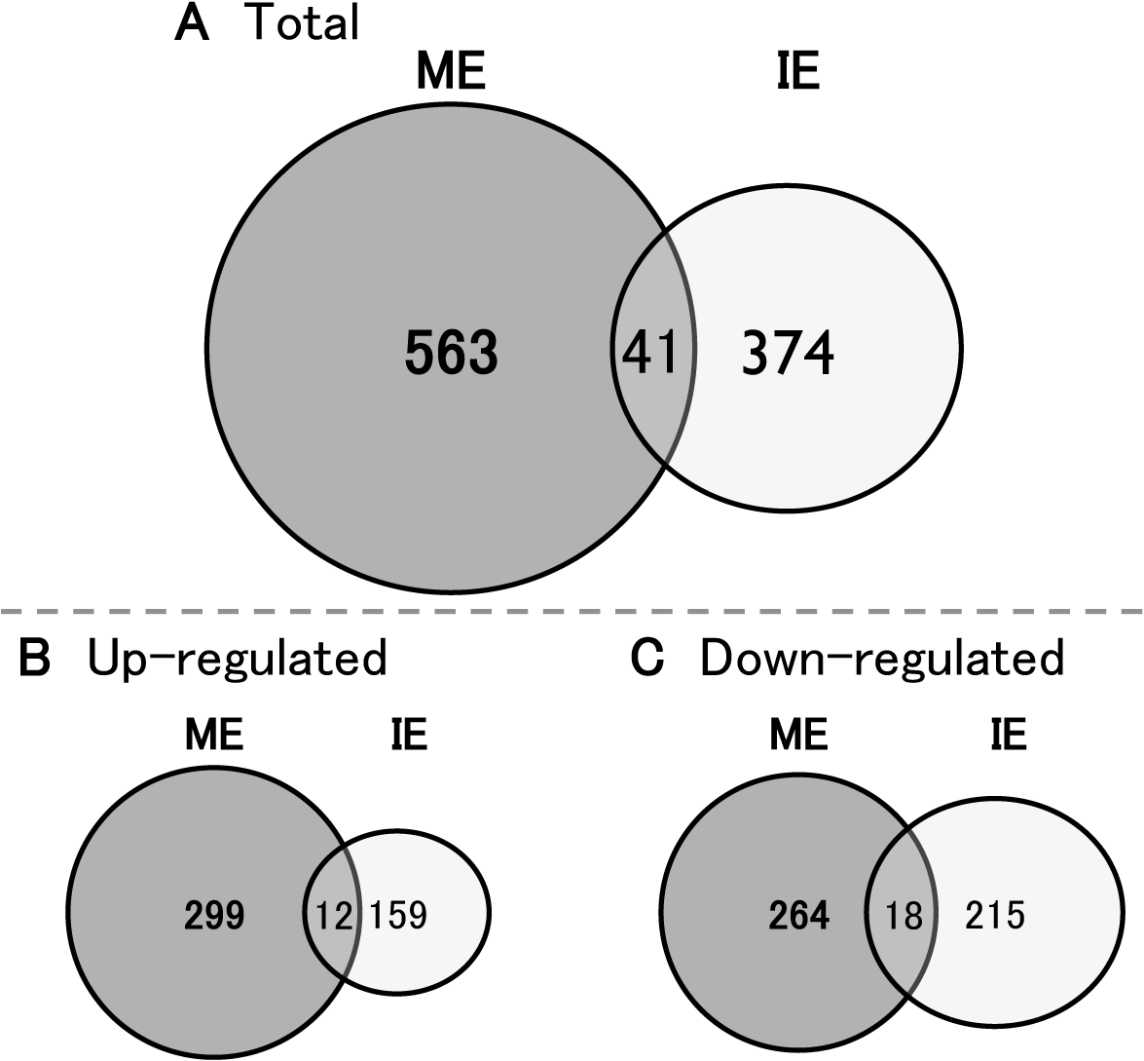
Venn diagram of differentially expressed genes in each condition. The total number of genes modified by ME (gray circles) and IE (white circles) (A), or up- (B) and down- (C) regulated genes in each condition are shown as Venn diagrams. The number in each circle indicates the selection of genes from the total microarray datasets within a defined fold range of greater than 1.5-fold and less than 0.75-fold versus sedentary (CONT).

Of 41,090 genes, more genes were regulated with ME (604 genes) than with IE (415 genes), and only 41 genes were commonly regulated with both exercise intensities (Fig 3A). The commonly regulated genes were composed of 11 oppositely regulated genes, 12 up-regulated genes and 18 down-regulated genes. Accordingly, 563 (up-regulated (up): 299, down-regulated (down): 264) and 374 (up: 159, down: 215) genes were specific to ME and IE, respectively (Fig 3B and 3C). It should be noted that the previously identified potential mediators (e.g., BDNF, IGF1, and VEGF) of exercise-induced neuronal change in the hippocampus were not identified in the gene sets from the present study. For detailed information on the changed gene expression profiles, readers are referred to the total gene expression data files at the NCBI GEO data repository (GSE 45813), submitted as part of this publication.

### Confirmation of gene expression

**Fig 4:**
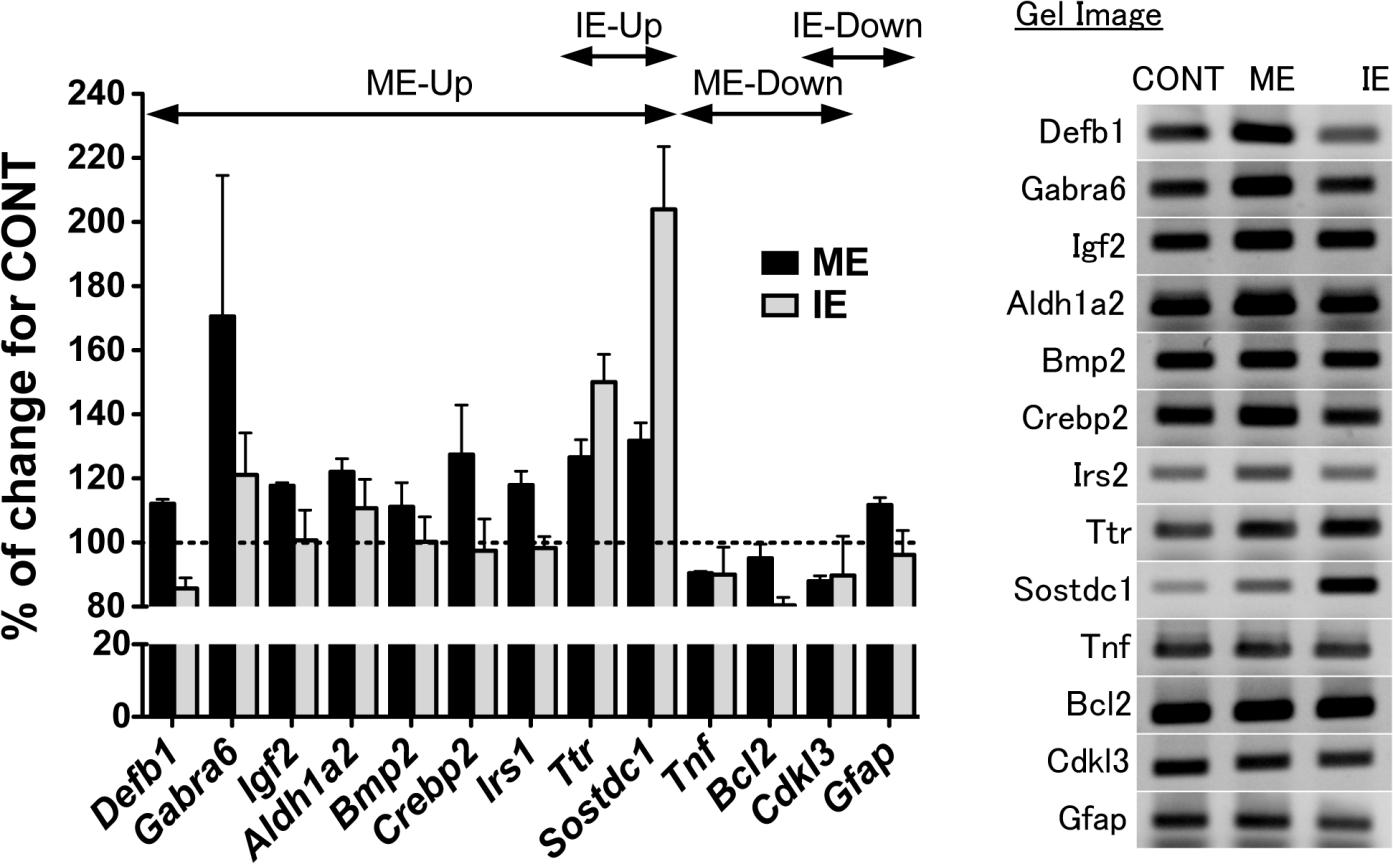
The mRNA expression profiles of 13 differentially expressed genes. Semi-quantitative RT-PCR validation of genes up- or down-regulated with ME or IE in the hippocampus. Each column indicates ME (black columns) or IE (gray columns), respectively. For each gene, the expression change is represented as the fold change in ME or IE relative to CONT. RT-PCR was performed as described in the Methods and the primers are detailed in S2 Table. Gel images on right-side show the PCR product bands stained with ethidium bromide.

To confirm the alterations in gene expression revealed with DNA microarray, 13 genes with annotated functions were randomly selected for RT-PCR analysis (as listed in S3 Table). These genes were selected for being potential factors in the regulation of hippocampal plasticity or because they were highly up-regulated or down-regulated with ME or IE. The mRNA expression profiles confirmed by RT-PCR (Fig 4) demonstrated that the DNA microarray data could be validated using appropriately designed primers and RT-PCR.

### Biological function of genes annotation with Ingenuity Pathway Analysis

**Fig 5:**
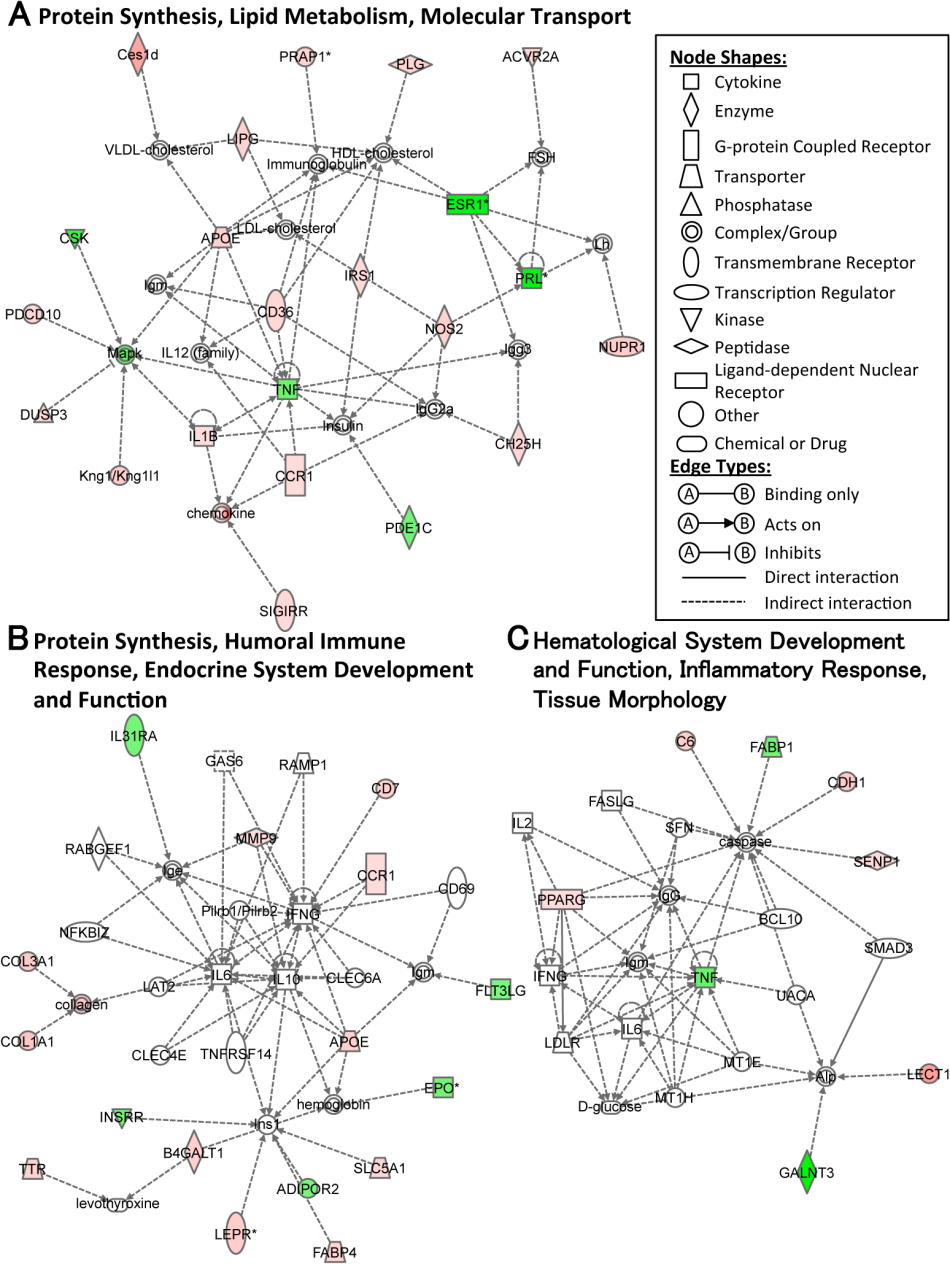
The top three gene-networks in response to mild exercise. Network analyses of the genes whose expression was modified with ME vs CONT are shown. Major functions of the networks are indicated in 1^st^ (A), 2^nd^ (B) and 3^rd^ (C) network. The score is the negative log of the *p* value and signifies the possibility of network-eligible genes within a network being clustered together as a result of chance. The biological relationship between two genes is represented as an edge (line), which is supported by at least one reference from the literature or a textbook. The intensity of the node color indicates the degree of up- (red) or down- (green) regulation. Nodes are displayed using various shapes that represent the functional class of the gene product.

**Fig 6:**
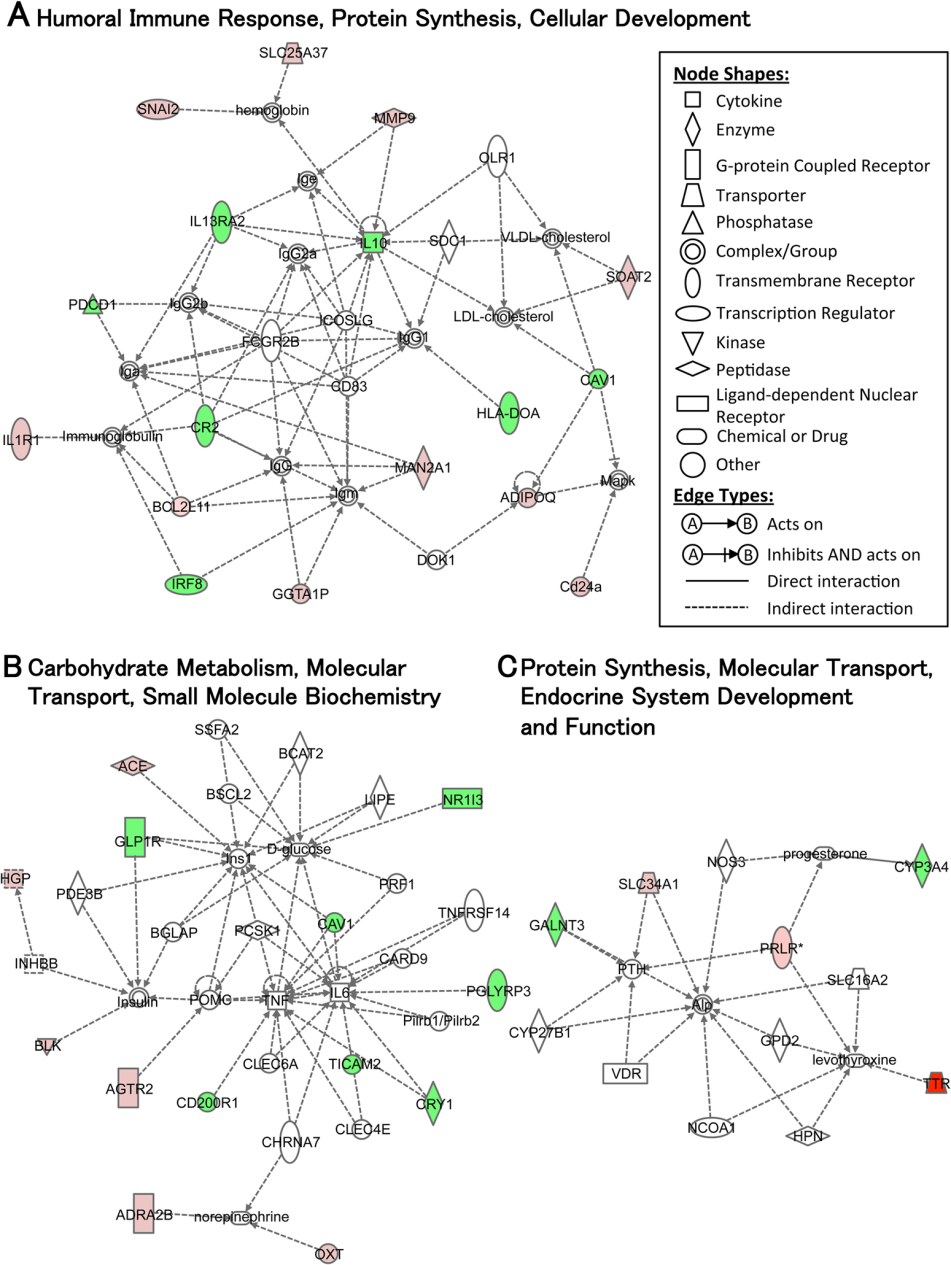
The top three gene networks in response to intense exercise. Network analyses of the genes whose expression was modified with IE vs CONT are shown. Major functions of the networks are indicated in 1^st^ (A), 2^nd^ (B) and 3^rd^ (C) network. Details are as indicated in Fig 5.

**Fig 7:**
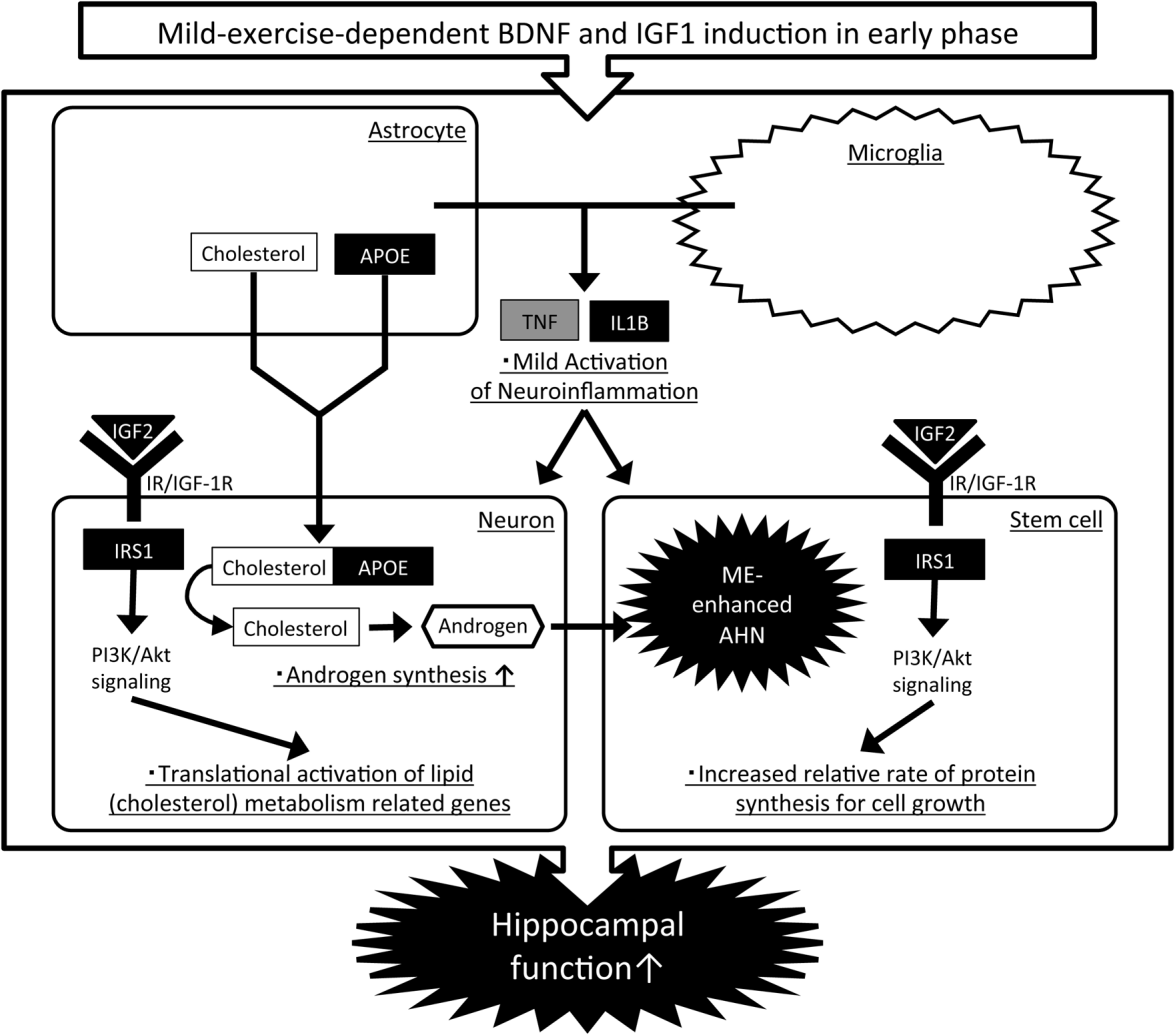
Hypothetical pathway related to ME-enhanced hippocampal function via AHN. Genes regulated by ME mapped to the pre-existing signaling pathway based on IPA-modified gene networks and previous literature. The names of up-regulated genes are written in white characters on a black background, those of down-regulated genes are written in black characters on a gray background, and those of unaltered genes are written in black characters on a white background. The genes and pathways related to lipid metabolism (cholesterol-trafficking), protein synthesis and inflammatory response that were up-regulated by ME might be a key to the process of enhancing hippocampal function through increased AHN.

When data is uploaded from the microarray for IPA, genes of which the detailed function remains unknown are excluded. In our case, the IPA narrowed down the genes detected by microarray to 231 (up: 129, down: 102) and 166 (up: 78, down: 88) for ME (S4 Table) and IE (S5 Table), respectively. A functional annotation of the genes is summarized in Table 1. The top three gene networks were also constructed on the basis of the known functions and interconnectivity of altered genes (Figs 5 and 6). In addition, we analyzed the molecular connection between each gene based on previous literature to gain further insight in order to characterize the molecular behavior regulating AHN with ME (Fig 7; IE data not shown).

**Table 1:** Top five gene functions modified with mild exercise (ME) or intense exercise (IE).

ME	IE
**Disease and Disorders**	
Cardiovascular Disease (39)	Renal and Urological Disease (29)
Organismal Injury and Abnormalities (47)	Organismal Injury and Abnormalities (40)
Renal and Urological Disease (8)	Cardiovascular Disease (41)
Cancer (89)	Connective Tissue Disorders (32)
Inflammatory Response (43)	Immunological Disease (30)
**Molecular and Cellular Functions**	
Lipid Metabolism (64)	Lipid Metabolism (30)
Small Molecule Biochemistry (73)	Molecular Transport (29)
Vitamin and Mineral Metabolism (43)	Small Molecule Biochemistry (40)
Drug Metabolism (25)	Cell-To-Cell Signaling and Interaction (47)
Molecular Transport (61)	Cellular Movement (26)
**Physiological System Development and Function**	
Endocrine System Development and Function (38)	Cardiovascular System Development and Function (40)
Embryonic Development (39)	Connective Tissue Development and Function (24)
Organ Development (38)	Embryonic Development (46)
Organismal Development (54)	Organ Development (40)
Reproductive System Development and Function (32)	Organismal Development (48)

Top 5 functional categories significantly altered (p<0.05) by each microarray combination. The number in parentheses is the gene number classified into the category. Mild exercise (ME): categories changed by mild-intensity exercise, Intense exercise (IE): categories changed by high-intensity exercise. The groups derived from Ingenuity Pathway Analysis (IPA) are categorized into disease and disorders, molecular functions and physiological system development and function.

Although genes regulated with ME were associated with a wide range of functions (Table 1), the majority were related to protein synthesis, lipid metabolism, molecular transport and the endocrine system. This trend was markedly reflected in the top three modified gene networks. For example, the 1^st^ (Fig 5A; score = 41) and 2^nd^ (Fig 5B; score = 26) networks for ME included these functions. Inflammatory/immune response was also supported by the 2^nd^ and 3^rd^ networks (Fig 5C; score = 12). For IE, the majority of genes were related to immune response, protein synthesis, cellular development and molecular transport supported by the IPA-based functional classification (Table 1) and the top three modified molecular networks (Fig 6A–6C).

## Discussion

In this study, we tested the effect of six weeks of mild (ME) treadmill running training on CORT level and AHN, and delineated the transcriptomic profile through a comparison with that of intense (IE) treadmill running training to delineate the mechanism associated with the reason why ME is more beneficial for improving spatial memory than is IE. Similar to our past study [5], only IE had the higher CORT level than in the CONT. Immunohistochemical results found that ME but not IE, enhanced AHN, especially the promotion of neuronal maturation of newly born cells. DNA microarray analysis showed that ME altered some novel AHN regulators, which are related to lipid metabolism (APOE), protein synthesis (IGF2, IRS1) and inflammatory response (IL1B, TNF). In contrast, these transcriptomic features were not confirmed in IE. These results support the hypothesis that effective improvement of AHN is caused by only long-term of ME having almost no running stress, and reveal a gene inventory that will aid in unraveling the molecular pathway involved in ME-induced adaptations.

### Exercise-intensity-dependent effects on body weight, plasma corticosterone and hippocampal neurogenesis

Our initial findings confirmed that body weight decreased with exercise regardless of its intensity (see results section), and that IE, but not ME, led to elevated CORT levels compared with CONT (Fig 1). The result is in agreement with our previous study showing that ME improves spatial memory without the influence of the exercise stress that accompanies IE [5]. In both studies, the exercised rats were habituated to the treadmill running at respective intensities using the electric shock, and rats trained almost spontaneously without the shocks after the habituation, as also shown in our previous studies [8,50]. The bad runners – an unsuited individual for intensity dependent change in AHN and hippocampal transcriptome – were excluded basing on the studies [8,50] in this case. Forced treadmill running exercise has been generally considered as a treatment that includes some stress because of studies indicating that the exercise, rather than voluntary exercise using running wheel, caused an increase in the CORT level in the serum [60,61]. In contrast, our study showed no change in the CORT level under ME. This result suggests that ME training in treadmill can be an exercise model barely containing the running stress but also that the potential adverse impact derived from the forcible manner of running and the shock could be mostly, but not completely, negated under the condition. It should be noted that even wheel running sometimes causes adrenal hypertrophy and hypercorticosteronemia [25], implying, “Exercise is accompanied by stress”; any of exercise includes some stress in its beneficial effects. Exercise stress can be of two types. One is specific probably via in part by the afferent signals from contracting muscles, tendon and joints. The other is non-specific caused by the environment, initial electrical shocks and emotional stressors, and so on. Thereby, chronic exercise manner or a certain term of running habituation would be fruitful in order to maximize positively the physiological effects, while minimizing the negative (non-specific) stress, through the successful stress adaptations suggested by Selye [62]. Indeed, in our earlier study for establishing the running stress model on the treadmill, we used the rats who learned running for a week, and successfully determined exercise with and without increase in lactate and stress hormones as a mild and moderate intensity exercise, based on the LT [8]. Moderate intensity was determined at 25 m/min for rats, and furthermore, we called it as just above the LT. This study has employed a more hard intensity (40 m/min) in IE training to highlight the effects of moderate intense exercise, and demonstrated that the exercise can be definitely considered a stressful condition as shown by the hypercorticosteronemia. Since the IE rats received a slight amount of additional electric shocks to keep their motivation for running comparing with the ME rats (see details in the Materials and Methods section), the fact that they might have been exposed to a greater stress associated with the shock cannot be ruled out. However, the gentle handling, a potential strategy to prevent the prolongation of the shock-induced stress [49,63], in the preliminary rearing, and our careful and slight use of the shocks in accordance with previous studies [48–50] should minimize or counteract the stress to the level that has little effect on the result. Interestingly enough, past data highly support the fact that rats continuously exposed to escapable, but not inescapable, electric shock can adapt or cope the stressor with no excessive secretion of stress hormone even when the shock is greater than our case [46,47]. The shock in treadmill exercise is avoidable by maintaining the running unlike with the unescapable box covered shock grid. Hence, even if the IE rats slightly received the stress effects from the shocks, they could cope with the stressor, and dampen the aversive effects to the minimal level that have no impact on the stress response. In our previous study, even in the hard exercise performed for six weeks, there are the beneficial effects of exercise training such as increased activity of citrate synthase, a typical oxidative enzyme and a good indicator for endurance. Taken together, although the forcible exercise-specific stress including the beneficial training effects cannot be fully separated from the non-specific stress, this study could minimize the interfering effects, and provide an exercise-intensity model having different effects vis-à-vis the stress response.

With the current model, we identified that both exercise intensities led to increased DG volume (Fig 2A) and stimulated cell proliferation (Ki-67^+^ cells) (Fig 2B). Meanwhile, only ME significantly enhanced cell survival (BrdU^+^ cells) and neuronal maturation (BrdU^+^/NeuN^+^ cells) (Fig 2C and 2D). The range of results for total DG volumes (2.1 to 2.4 mm^3^) is similar to results from previous rat studies [64,65], and the total number of newborn cells in the whole DG was estimated by multiplying the total DG volume by the mean cell density in all sections. Thus, we believe that these data should be highly reliable and valid. These results suggest that ME, rather than IE, effectively boosts AHN throughout the maturation stage. A past study from Grégoire and co-workers has shown that exercise could stimulate all stages of AHN independently of the effects of other variables, such as environmental complexity and social context [66], and thus support our result with ME. Most importantly, these results are the first findings for a six-week, rather than two-week [23], exercise model.

Interestingly enough, the number of BrdU^+^ and BrdU^+^/NeuN^+^ cells in IE has no difference from that in CONT and in ME (Fig 2C and 2D). The result does not significantly recognize the IE-dependent effects on AHN, but implies an intermediate effect of IE on cell survival and neuronal maturation. Past studies have intensively investigated the influence of stress or CORT level on AHN, and reported that chronic stress suppresses AHN through the action of CORT [67], and have negative impact on hippocampal-dependent function, such as the induction of memory loss and depression-like behavior [68–71]. Given that IE is stressful condition in the presence of hypercorticosteronemia (Fig 1), as with the case of these studies, the IE condition-derived stress may exert an inhibiting effect on AHN, resulting in the suppression of exercise-induced AHN to the intermediate level. Specifically, most newly born cells may die or differentiate into astrocytes due to the negative effects of stress resulting from IE as not only ME but also IE significantly enhanced the cell proliferation (Fig 2B). As contrasted with the AHN modification by stress, stress response and stress-triggered behavioral impairment are regulated with the degree of AHN [72]. Furthermore, exercise having AHN-enhancing effects has been used as a strategy for encountering the stress-related deleterious effects, despite being open to debate [66]. Numerous studies have actually shown that continuous exercise training recovers the decrease in AHN, attenuates the depression-like behavior, and restores the MWM task performance to normal level after chronic stress or CORT-treatment [69–71]. These findings lead us to further hypothesis that IE may activate the stress response, and accelerate its dependent adverse impact through the suppression of AHN instead of enhancing stress tolerance. However, if the IE rats adapted to the intensity, resulting in the negation of stress effects, during a longer period of training, even the heavy exercise might increase the AHN to almost the same level as ME.

The exercise-intensity dependent regulation of AHN is supported by our recent study [23] and previous findings showing that he ME-like light exercise enhances AHN [73], while the IE-like exercise does not alter AHN [74]. Considering the fact that mature neurons with unique features of the morphology and the electrical activity are a possible contributor in memory formation [11–18] and that exercise, which increases the number of new mature neurons, improves spatial memory but not spatial learning [4], the increased number of surviving neurons by ME (Fig 2D) could plausibly lead to improved spatial memory. Further study to define the relationship between ME-induced changes in AHN and in spatial memory is required.

### Overview of the hippocampal transcriptome for ME and IE

We next compared the intensity-dependent gene expression profile of the hippocampus by performing a whole-genome DNA microarray in order to obtain new molecular insight of ME-induced increases in AHN. Results showed that a larger number of genes were differentially expressed with ME than with IE compared to an appropriate control (Fig 3). Interestingly, the gene inventory did not contain well-known AHN-related regulators in exercise such BDNF, IGF1 and VEGF [4,29,30–33]. Although a clear explanation of this result is difficult, the reasons may be associated with the difference in timing of the collection of hippocampal tissue after exercise, and the technical features of microarray analysis. In this study, we analyzed gene expression using microarrays two days after the last training session in order to observe the effects of chronic exercise on the hippocampal transcriptome without the influence of acute exercise effects. It should be noted that Huang et al. (2006) examined the effects of four weeks of treadmill exercise on the expression of BDNF in the rat hippocampus, and found that the BDNF synthesis was up-regulated 2 hours, but not 2 days, after the last training session [75]. The study indicated that exercise training could acutely enhance hippocampal BDNF synthesis, whilst the effects disappeared within 2 days; this strongly supports our microarray results, and suggests that ME may also increase BDNF synthesis at an earlier time point after the last training session. In addition, as microarray data reflected the transcriptomic changes in the hippocampus, it may be reasonable that IGF1 and VEGF, reported as essential mediators of exercise-induced AHN [31,32], are not included in our results due to their peripheral nature. However, that they remain unidentified in this study does not give us leave to ignore the contribution of these postulated factors in regulation of AHN with ME. In fact, ME-dependent gene induction of IRS1 (Fig 5A and S4 Table), a primary cytosolic substrate of both insulin and IGF1 receptors [76], indicates the possibility that upstream factors, such as IGF1, may affect the gene alteration resulting from ME.

### Features of transcriptomic changes for each exercise intensity

Functional and network analysis of microarray data indicates that each exercise intensity controls a gene cluster with an individual function (Figs 5 and 6, Table 1). For example, the top three gene networks of ME inferred by IPA showed that the ME modulated lipid metabolism, protein synthesis, molecular transport, and inflammatory/immune response. Conversely, IE regulated immune response, protein synthesis, cellular development, and molecular transport. While the relationship between exercise and hippocampal lipid metabolism is not yet well understood, some studies have suggested that exercise increases the rate of protein synthesis in the hippocampus [77], and that exercise [78] or chronic stress [79], which could also apply to IE-induced exercise stress, reduces or worsens inflammation and immune response, respectively. These facts support our results showing that genes associated with these functions were modified by ME or IE, respectively. To accomplish our goal in using DNA microarray, we subsequently focused on the process, networks and specific genes associated with AHN. Some of the noticeable gene alterations that we identified are discussed below according to their respective functions.

#### Lipid metabolism and molecular transport

ME significantly modulated the lipid metabolism-related genes including two crucial transporters of cholesterol (APOE) and fatty acid (FA) (CD36), which were found to be up-regulated (Fig 5A), whereas this process was not significant in IE. In the central nervous system (CNS), cholesterol is a major structural component of membranes and myelin sheaths [80]. Additionally, previous studies have shown that cholesterol in neurons becomes an important source for neuroplastic development and for the biogenesis of steroid hormones [80,81]. The major source of cholesterol in the CNS is *de novo* synthesis from astrocytes, but not circulation [80], and cholesterol transport to neurons is controlled with APOE, which is derived mainly from astrocytes in the CNS [82] as well as the hippocampus [83]. In past studies, APOE has been reported as a crucial regulator in the normal development of AHN [84] and synaptic structural plasticity [85]. Moreover, our current study identified that ME enhanced the synthesis of hippocampal dihydrotestosterone, which is derived from cholesterol in neurons [86], and increased AHN through androgenic mediation [23]. These studies indicate that APOE derived from astrocytes might play an important role in the trafficking of cholesterol, which is a source of ME-induced increased AHN via enhanced androgen effects, from astrocytes to neurons. In addition to cholesterol, FA, an intracrine or paracrine neuromodulator of AHN [87], could be associated with the exercise-dependent beneficial effects of improving hippocampal function and AHN. A recent study reported that exercise-induced cognitive gain and enhanced AHN was impaired if the synthesis of FA was blocked [88]. Though the contribution of CD36 to FA transport remains unclear, the increased expression of CD36 gene with ME may serve to increase AHN and cognitive gain by enhancing the function of FA. Collectively, the induction of cholesterol- and FA-traffic-related genes (APOE, CD36) by ME might contribute to the exercise-intensity-induced increment in AHN. Notably, astrocyte-derived APOE might influence androgen synthesis in neurons, resulting in the increased AHN through the androgenic effect in ME.

#### Protein synthesis and cellular development

Both exercise intensities appear to principally regulate protein synthesis, which is highlighted in the 1^st^ and 2^nd^ ME networks (Fig 5A and 5B) and the 1^st^ IE network (Fig 6A). The 1^st^ IE network also reveals an intensity-dependent modulation of cellular development. With ME, modulation of this process may occur via the up-regulated IRS1 gene (Fig 5A and S4 Table). Generally, IRS1 stimulated by insulin or IGF1 leads to the induction of a downstream PI3K-Akt or MAPK/ERK signaling cascade, which increases protein synthesis via translational activation to cellular growth [76,89] and promotes cell survival [90]. In our case, microarray results identified the up-regulation of IGF2 gene expression (Fig 4 and S4 Table, but not included in the top ME network), an upstream factor of IRS1 [91,92], and the suppression of MAPK gene expression (Fig 5A) with ME. Hence, the induction of the IRS1 gene might be regulated not only by insulin or IGF1, but also by IGF2, and may activate PI3K-Akt signaling rather than MAPK/ERK signaling. In the hippocampus, IRS1 is found in neurons [93]. Although whether it is expressed in adult neural stem cells remains unknown, upstream regulators of IRS1, such as the IGF1 receptor, are located in the stem cells [33]. Following these findings, protein synthesis and/or cell survival via the induction of the IRS1 gene might be activated not only in hippocampal neurons but also in the stem cells. In contrast, IE suppressed the expression of caveolin-1 (CAV1) (Fig 5B and 5C), a structural protein in membrane lipid rafts that is involved in synaptic development/stabilization and neuronal signaling. Growing evidence demonstrates that CAV1 accelerates NMDA-, BDNF- and insulin-mediated signaling cascades [94,95]. BDNF signaling is also indispensable for new protein synthesis [96] and cellular growth [97], thus the suppression of CAV1 by IE might weaken these processes, resulting in the exercise-dependent effects on AHN being dampened, as seen in Fig 2C and 2D. Thus, ME-induced AHN could occur with the induction of protein-synthesis-related genes (IGF2, IRS1) in neurons or neural stem cells, while exercise-induced stress, like that resulting from IE, attenuates positive effects with the down-regulation of the protein-synthesis- and cellular-development-related gene (CAV1) in the plasma membrane.

#### Inflammatory and immune response

Current consensus leans toward the idea that the overall effect of inflammation in the hippocampus is determined by various types of inflammatory cytokines, prostaglandins and neurotrophins, which are mainly secreted from microglia or astrocytes, and exhibit an inverted U-shaped pattern for neural plasticity and function [79]. Note that this idea is infiltrating the field of study examining the effects of exercise in hippocampal neuroplasticity [74]. The current study detected several pro- and anti-inflammatory cytokines with both exercise intensities. For example, ME up-regulated the pro-inflammatory cytokine IL1B within the 1^st^ network while suppressing the pro-inflammatory cytokine TNF within the 3^rd^ network (Fig 5A and 5C). It is notable that the excessive activation or blockade of IL1 signaling causes impairment of memory, whereas a mild activation improves memory function [98]. Further, TNF has been assumed as a negative regulator of the neuronal differentiation of AHN [99] and hippocampal function [100]. By combining these studies with the present results for AHN (Fig 2), it becomes evident that the moderate level of pro-inflammatory activation in ME might be related to enhanced hippocampal neuroplasticity including AHN. Conversely, IE may induce an excessive inflammatory response through the down-regulation of the anti-inflammatory cytokine IL10, and the up-regulation of the pro-inflammatory cytokine IL1 receptor IL1R1 (Fig 6A). If the suppressed genes with IE could be prevented by modification of the exercise condition, IE could result in the development of AHN and hippocampal function. Taking these results together, we speculate that the degree of hippocampal inflammatory response with each exercise condition, as observed through their respective gene expression changes, is monitored by microglia or astrocytes and leads to a crucial induction or inhibition of AHN.

### Hypothetical pathways associated with ME-induced hippocampal neuroadaptations

Based on the top molecular networks (Fig 5) and the published findings on genes confirmed in this study, here we outline the predictable molecular mechanisms concerned with ME-induced AHN development that might contribute to cognitive gain (Fig 7). The factors and pathways associated with cholesterol transport (APOE), protein synthesis (IGF2, IRS1) and inflammatory response (IL1B, TNF) seem to be potential regulators in ME-dependent adaptations. As mentioned above, with ME, APOE derived from astrocytes might contribute to the biosynthesis of androgen in neurons by supplying the source (cholesterol). Androgen affects stem cells in the hippocampus and promotes their survival and development, as shown in our previous study [23]. On the other hand, the up-regulation of protein-synthesis-related genes (IGF2, IRS1) could potentiate the androgen synthesis in neurons through translational activation of genes related to the synthetic process and/or contribute toward the cell growth of stem cells by increasing the relative rate of protein synthesis and cell survival. Moreover, IL1B and TNF from microglia or astrocytes may cause a moderate inflammatory response in neurons or stem cells, enhancing AHN and hippocampal function. As stated above, these effects can be potentially affected with well-known regulators such as BDNF and IGF1 (see the section entitled, “Overview of the hippocampal transcriptome for ME and IE”).

### Limitation and future challenges

This study has examined the exercise-intensity-dependent effects on AHN and hippocampal genome-wide transcriptome in a forced treadmill exercise training model, and indicated the differences between ME and IE. The model, however, might have certain non-specific stress derived from the electrical shocks for the forcible exercise. Thus, the possibility that the confounding factors affect our outcomes presented here cannot be completely negated. In addition, we have potentially identified some of the contributors involved in ME-induced increment of AHN, and described a hypothetical mechanism focused on these contributors. However, since the present study could not test the link between the identified contributors and the ME-induced adaptations, it would be rather premature to define their functional role in ME, because of methodological complexities. Therefore, subsequent studies will require a foundation of experimental evidence whether or not these genes is involved with the ME-induced adaptation. Functional genomics approaches such as generation of transgenic animals, gene knockdown techniques (i.e. siRNA) or pharmacological methods involving use of specific inhibitors may allow us to perform these new experiments and analyses. Furthermore, it is crucial that, over time, we piece together the validated role of each individual regulator in order to clarify the main pathway involved in ME-dependent hippocampal adaptations, and the present results provide one part of that extensive puzzle. These functional approaches to characterizing the role of genes, including those which we have identified here, will facilitate a deeper understanding how ME modulates hippocampal neuroadaptation.

## Conclusion

In conclusion, our results manifested that six weeks of mild (ME), but not of intense (IE), treadmill running training with minimizing running stress is effective in enhancing AHN, and clarified the ME-specific induction of genes that might have positive effects in regulating AHN. Notably, genes related to lipid metabolism (APOE), protein synthesis (IGF2, IRS1) and inflammatory response (IL1B, TNF) could be important clues for understanding the molecular mechanism of ME-enhanced AHN. Although further study is required to examine the relationship between these findings and hippocampal function, the ME-specific gene inventory is information essential to elucidating why ME, but not IE, contributes to improved spatial memory in our rat model.

## Supporting Information

S1 Table Exercise training protocol. (XLSX)

S2 Table Comparison of RNA quality control parameters. (XLSX)

S3 Table Primer combinations used for semi-quantitative RT-PCR. (XLSX)

S4 Table Complete list of genes uploaded to IPA for ME. (XLSX)

S5 Table Complete list of genes uploaded to IPA for IE. (XLSX)
