# Correction: Long-Term Mild, rather than Intense, Exercise Enhances Adult Hippocampal Neurogenesis and Greatly Changes the Transcriptomic Profile of the Hippocampus

**DOI:** 10.1371/journal.pone.0133089

**Published:** 2015-07-13

**Authors:** Koshiro Inoue, Masahiro Okamoto, Junko Shibato, Min Chul Lee, Takashi Matsui, Randeep Rakwal, Hideaki Soya

There are errors in Fig 3 pertaining to the number of genes identified by microarray. The authors have provided a corrected version here.

**Fig 3:**
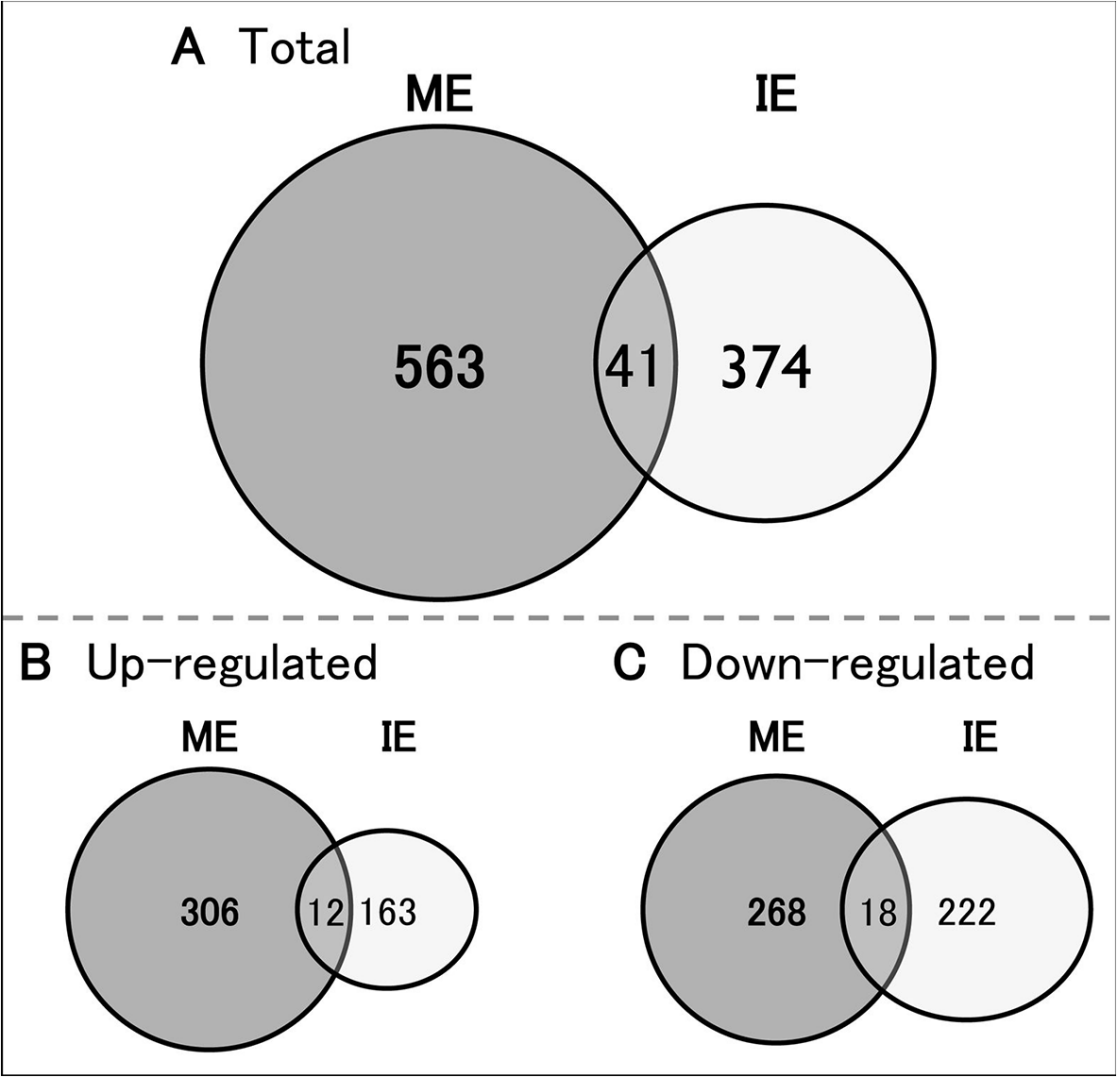
Venn diagram of differentially expressed genes in each condition. The total number of genes modified by ME (gray circles) and IE (white circles) (A), or up- (B) and down- (C) regulated genes in each condition are shown as Venn diagrams. The number in each circle indicates the selection of genes from the total microarray datasets within a defined fold range of greater than 1.5-fold and less than 0.75-fold versus sedentary (CONT).

In the “Overview of the hippocampal transcriptome” subsection of the Results, the third sentence should read: Accordingly, 574 (up-regulated (up): 306, down-regulated (down): 268) and 385 (up: 163, down: 222) genes were specific to ME and IE, respectively (Fig 3B and 3C).
